# Population-level consequences of complementary sex determination in a solitary parasitoid

**DOI:** 10.1186/s12862-015-0340-2

**Published:** 2015-05-30

**Authors:** Jetske G de Boer, Martien AM Groenen, Bart A Pannebakker, Leo W Beukeboom, Robert HS Kraus

**Affiliations:** Evolutionary Genetics, Centre for Ecological and Evolutionary Studies, University of Groningen, P.O. Box 11103, 9700 CC Groningen, The Netherlands; Laboratory of Entomology, Wageningen University, Droevendaalsesteeg 1, 6708 PB, Wageningen, The Netherlands; Animal Breeding and Genomics Centre, Wageningen University, Droevendaalsesteeg 1, 6708 PB, Wageningen, The Netherlands; Laboratory of Genetics, Wageningen University, Droevendaalsesteeg 1, 6708 PB, Wageningen, The Netherlands; Department of Biology, University of Konstanz, 78457 Konstanz, Germany; Max Planck Institute for Ornithology, Department of Migration and Immuno-Ecology, Am Obstberg 1, 78315 Radolfzell, Germany

**Keywords:** Hymenoptera, Whole genome sequencing, Biological control, Inbreeding depression, Mating system

## Abstract

**Background:**

Sex determination mechanisms are known to be evolutionarily labile but the factors driving transitions in sex determination mechanisms are poorly understood. All insects of the Hymenoptera are haplodiploid, with males normally developing from unfertilized haploid eggs. Under complementary sex determination (CSD), diploid males can be produced from fertilized eggs that are homozygous at the sex locus. Diploid males have near-zero fitness and thus represent a genetic load, which is especially severe under inbreeding. Here, we study mating structure and sex determination in the parasitoid *Cotesia vestalis* to investigate what may have driven the evolution of two complementary sex determination loci in this species.

**Results:**

We genotyped *Cotesia vestalis* females collected from eight fields in four townships in Western Taiwan. 98 SNP markers were developed by aligning Illumina sequence reads of pooled DNA of eight different females against a *de novo* assembled genome of *C. vestalis*. This proved to be an efficient method for this non-model species and provides a resource for future use in related species. We found significant genetic differentiation within the sampled population but variation could not be attributed to sampling locations by AMOVA. Non-random mating was detected, with 8.1% of matings between siblings. Diploid males, detected by flow cytometry, were produced at a rate of 1.4% among diploids.

**Conclusions:**

We think that the low rate of diploid male production is best explained by a CSD system with two independent sex loci, supporting laboratory findings on the same species. Fitness costs of diploid males in *C. vestalis* are high because diploid males can mate with females and produce infertile triploid offspring. This severe fitness cost of diploid males combined with non-random mating may have resulted in evolution from single locus CSD to CSD with two independent loci.

**Electronic supplementary material:**

The online version of this article (doi:10.1186/s12862-015-0340-2) contains supplementary material, which is available to authorized users.

## Background

Sex determination systems that initiate differentiation between males and females are highly diverse across the animal kingdom, with examples of temperature dependent sex determination (e.g. in reptiles), heterogametic males in mammals and heterogametic females in birds, and a range of other (genetic) mechanisms in amphibians, reptiles and insects [1-4]. Transitions among sex-determining mechanisms have occurred repeatedly across the animal kingdom [1,5], and genetic experiments on the nematode *Caenorhabditis elegans* confirm that sex determining mechanisms are indeed extremely labile [6]. Despite growing knowledge on *how* sex determination mechanisms can evolve, the evolutionary drivers of these mechanisms are understood less well [4,7,8].

The insect order Hymenoptera is interesting in this respect because of the intricate relationship between mating structure and sex determination [9,10]. All sexually reproducing Hymenoptera are haplodiploid, with haploid males developing from unfertilized haploid eggs, while females develop from fertilized diploid eggs. Sex is not determined by fertilization alone, however, and *diploid* males can develop from fertilized eggs that are homozygous at a sex locus under a system called complementary sex determination (CSD) [11]. Heterozygosity at the sex locus leads to female development. The presence of diploid males, suggesting CSD, has been demonstrated in a variety of parasitoid wasps, ants, solitary and social bees and wasps [9,12]. Genetic support for the sex locus is lacking in most of these species due to the limited availability of genetic markers and the difficulty to make controlled inbred crosses in the laboratory, particularly for social species [12]. An exception is formed by bumblebees, in which the sex locus has been mapped [13-15], and honeybees, in which the *csd* gene has been mapped, cloned and sequenced [13,14,16,17].

In terms of genetics, CSD resembles the self-incompatibility (SI) breeding system of plants, and the major histocompatibility (MHC) locus in vertebrates because heterozygotes in all three systems have a fitness benefit [18], and high allelic diversity can be maintained through frequency dependent selection. The fitness costs of CSD are potentially even more severe than those associated with SI and MHC because the fusion of gametes with incompatible sex alleles effectively leads to post-zygotic mortality. Diploid males resulting from sex locus homozygotes have near-zero fitness because they are commonly sterile or unviable, and they are produced at the expense of fertile daughters. It is thus expected that populations with CSD experience strong selection on reducing the costs associated with the production of diploid males [7]. In species in which CSD is maintained, selection may act to reduce levels of inbreeding or the cost of diploid males. For example, in *Bracon hebetor* and *Bombus terrestris*, behavioural adaptations reduce the level of mating between siblings [19-21], and in *Euodynerus foraminatus* and *Cotesia glomerata*, diploid males are able to sire diploid daughters, presumably by producing haploid sperm [22,23]. Alternatively, selection may lead to the evolution from CSD to mechanisms of sex determination with fewer or no diploid males. Interestingly, within the Hymenoptera only one other mechanism of sex determination is currently supported experimentally: maternal imprinting in the parasitoid *Nasonia vitripennis* [24,25].

We previously showed that CSD in *Cotesia vestalis* (Braconidae) is likely caused by two unlinked sex loci, based on the rate of diploid male production in laboratory crosses [26]. The genus *Cotesia* is polymorphic for CSD variants as well as for other life history parameters, which makes it a valuable system to study evolutionary drivers of sex determination [10]. *Cotesia vestalis* is a solitary Eurasian parasitoid [27,28], which has been introduced for biological control of diamondback moth worldwide [29]. Solitary parasitoids are expected to have lower rates of inbreeding than gregarious parasitoids because siblings emerge from the same host in the latter [30]. This makes it surprising that *C. vestalis* has evolved a second sex locus whereas the closely related *C. glomerata* has maintained single locus CSD (sl-CSD) despite high inbreeding rates in nature [31-33]. This raises the question what has driven the evolution of two-locus CSD in *C. vestalis.* Here, we investigate the hypothesis that non-random mating in nature may have selected for the evolution of two-locus CSD. We expect that under natural conditions, *C. vestalis* (1) produces diploid males at a low rate; (2) shows non-random mating; and (3) has little population structure. Because very few molecular markers were available for *C. vestalis*, we generated a superior genomic resource by sequencing the entire genome of *C. vestalis* by Illumina technology. After *de novo* assembly we developed a set of single nucleotide polymorphism (SNP) assays for population-wide studies in this species*.* This approach also provides a backbone for similar studies in closely related species that may lead to further comparative studies of CSD.

## Methods

### Collection of field samples

*Cotesia vestalis* (Haliday) was collected in Western Taiwan in February of 2008. Two small non-commercial vegetable gardens with apparent diamondback moth infestations were selected in each of four townships: Luhzu (Kaohshiung county), Sihu (Changhua county), Shanhua (Tainan county) and Shingang (Chiayi county). A variety of crops was grown in these fields, including different types of host plants for *Plutella xylostella* L. (diamondback moth larvae), such as Chinese cabbage, broccoli, stem cabbage, kohlrabi and cauliflower. In most fields, other, non-host crops were grown as well, or were bordering the cabbage field, such as tomato, corn, sweet pepper, papaya, beans and onions.

In each field, 15 to 24 host plants were randomly selected from which we collected all *C. vestalis* cocoons and diamondback moth larvae (first to third instar) (Additional file 1: Table S1). *Cotesia vestalis* cocoons were directly transferred to small Petri dishes (5 cm diameter) with some droplets of honey and kept in the refrigerator until transportation to the Netherlands (a maximum of 2 weeks). Diamondback moth larvae were transferred to plastic containers (18x13x6cm) with a square hole in the lid covered with fine mesh for ventilation. Larvae were provided with fresh cabbage leaves every other day for one week at 20-30°C. By then, most larvae had either pupated or a *C. vestalis* cocoon had emerged. After one week, *C. vestalis* cocoons were collected into small Petri dishes and kept in the refrigerator as described above; remaining material was discarded. All *C. vestalis* cocoons collected and/or reared from a single plant were kept separately.

After transportation to the Netherlands, *C. vestalis* cocoons were kept at room temperature in a laboratory at the University of Groningen. Petri dishes were checked daily for adult emergence and emerged wasps sexed: Females were identified by the presence of an ovipositor with a stereomicroscope. Males and females were then frozen at −20°C for analysis of ploidy.

### Analysis of ploidy

To assess the consequences of CSD in a natural field population, we analysed ploidy levels of approximately 2/3^rd^ of all *C. vestalis* males, including all males of fields 1, 3, 4, 6 and 7 and a random subset of males from fields 2, 5 and 8 (Additional file 1: Table S2). We followed methods described previously for *Cotesia* parasitoids [34,35]. In short, the head of an individual wasp was ground in 0.5 ml ice-cold Galbraith buffer [36] with a Dounce tissue grinder. The cell suspension was sieved (40 μm) and stained with propidium iodide (25 μg per sample). DNA content of 2500 nuclei from head tissue was measured per wasp on an Epics® XL™ flow cytometer (Beckman Coulter, Brea, CA). DNA histograms were compared to those of known haploid males and diploid females to classify ploidy levels as haploid, diploid or unknown. After the head of a wasp was cut off, the rest of the body was placed in 96% EtOH and kept at 4°C for genotyping.

### SNP detection, assay development and genotyping

#### *De novo* assembly and the *Cotesia vestalis* reference genome

No genome information was available for *C. vestalis*. In order to build a draft reference genome and to develop SNP assays, we sequenced the entire genome of *C. vestalis* on a single lane of paired-end sequences (2x100 bp) on an Illumina HiSeq 2000 (Illumina Inc., U.S.A.) instrument. The SNP discovery panel consisted of eight *C. vestalis* females, one from each field, from a randomly selected plant. DNA from the entire bodies was extracted as a pool using Qiagen’s DNeasy kit following the protocol for tissue DNA with an extended incubation time in buffer ATL (3 h at 56°C). A single genomic library with an insert size of approximately 300 bp was prepared using the Illumina Sample Preparation Kit and sequenced for 100 cycles with the Illumina HiSeq 2000 (paired-end) at the UMCG Sequencing Facility in Groningen, The Netherlands. The raw data files from the sequencing instrument are deposited in the NCBI short read archive under accession number SRP058413 [79].

Before assembly, Illumina reads were trimmed using an in-house Perl script that trims the sequence as soon as two consecutive bases have a quality score lower than 20. Reads that after trimming had a length smaller than 50 bp were removed from the analysis. To obtain *C. vestalis* sequence contigs to be used as a pseudo-reference genome, we performed a *de novo* assembly on the 133 million 100 bp reads using SOAPdenovo version 1.05 [37]. The assembly was done using a k-mer size of 45 and k-mers that were seen only once were removed (option –d). After contig construction, scaffolding was performed using intra-scaffold closure (option –F) and a minimum length for scaffolding of 50 bp. The total size of the assembly was 152 Mb with a contig N50 size of 761 bp and a scaffold N50 size of 2400 bp. With an estimated size of the *Cotesia vestalis* genome of 190 Mb (estimated with flow cytometry by J.G. de Boer, unpublished data) this suggests that our assembly covers around 80% of the *Cotesia vestalis* genome. We subsequently concatenated all scaffolds and unassembled contigs into a single artificial chromosome to be used as a reference genome for SNP identification. Assembled scaffolds and contigs have been deposited in Dryad [80]. Contigs and scaffolds were concatenated at random using a spacer sequence of 150 N’s.

#### Alignments of reads and SNP identification

Individual paired-end reads were aligned against the artificial *Cotesia vestalis* reference genome obtained from the *de novo* genome assembly using BWA [37,38]. The resulting BAM file was then used for the identification of putative SNPs using SAMtools and varFilter from the samtools.pl utility [39]. We only considered nucleotide substitutions and ignored small indels. SNPs were filtered that had a mapping quality higher than 20, a minimum read depth of 3 and a maximum read depth of 90 (3x the average read depth, a strategy to avoid orthologous SNPs, e.g. in multi copy genes [40,41]).

#### SNP selection and assay development

##### Selection

From our list of putative SNPs across the *C. vestalis* genome, we selected 100 SNPs for genotyping assay development. We first selected the 200 largest scaffolds; they varied in length from 17-58Kb and contained a total of 7,878 SNPs. We then removed SNPs with a minor allele frequency (MAF) <0.2, SNPs that had another SNP within 50 bp up- or downstream, and SNPs with more than 2 alleles. The remaining SNPs were binned in MAF bins of 0.2-0.3 (1,908 SNPs), 0.3-0.4 (1,605 SNPs) and 0.4-0.5 (1,156 SNPs). Per MAF bin, SNPs were ranked by SNP quality score. We then selected the SNPs with the highest quality scores, picking 20 SNPs with a MAF between 0.2-0.3 and 40 each with a MAF between 0.3-0.4 and 0.4-0.5, all on different scaffolds. All selected SNPs had a quality score of more than 200 (based on SAMtools), and an average read depth of 61. High-throughput genotyping assays based on allele-specific forward primers were developed for these 100 SNP sequences at KBioscience (now LGC Genomics, Hoddesdon, U.K.). Kompetitive allele specific PCR (KASP) technology has been used successfully on a wide range of organisms, including plants, vertebrate and invertebrate animals, and, for plants, compares favourably to chip-based methods such as GoldenGate in terms of accuracy and cost [42].

##### Validation

Because the entire bodies of *C. vestalis* females were used in the DNA pool of the SNP discovery panel, we selected another female from the same plant in each field to validate the selected SNPs. DNA was extracted from the entire body using Qiagen’s DNeasy kit as described above. These eight females were subsequently genotyped with the KASP assays at KBioscience to establish whether the selected SNP loci were polymorphic.

##### Genotyping

To assess *C. vestalis* mating structure in the Taiwanese field population, we genotyped one female per plant of each field at 98 SNPs that were successfully developed into KASP assays. In addition, all males that were identified as diploid with flow cytometry were genotyped, males identified as haploids were not genotyped to save costs. DNA extractions were performed on entire parasitoid wasp bodies at KBioscience with the Kleargene protocol.

### Data analysis

All wasps and all loci were included in the analyses because very few genotypes were missing (on average 2.5% of loci were missing per wasp). On average 3.5% of genotypes was missing per locus with a maximum of 11 out of 139 wasps failing to genotype at any one locus.

We first used Structure 2.3.3 [43,44] to investigate genetic clustering within the complete data set without prior information on sampling locations. We performed 10 independent runs each for *K* = 1 to *K* = 8 (where K is the number of clusters), using the admixture ancestry model with correlated alleles, a burn-in length of 50,000 steps and 100,000 MCMC steps. Evanno’s delta *K* method [45] was used to assess *K*.

The largest cluster (76 individuals) inferred by Structure was subsequently used to examine the SNP loci in terms of deviations from linkage and Hardy-Weinberg equilibria because we generated the markers *de novo* and had no information on their suitability as population genetic markers. The other clusters were considered too small for robust statistical analysis. Statistical comparison of our data to expectations under linkage equilibrium were done with likelihood ratio tests with 16,000 permutations and setting the number of initial conditions for expectation maximization to 5 [46] in Arlequin 3.5.1.3 [47]. To account for the large number of statistical tests in this pairwise procedure, p-levels for statistical significance were adjusted with a sequential Bonferroni correction for multiple comparisons [48]. Deviations from Hardy-Weinberg equilibrium (HWE) in the largest cluster were also tested in Arlequin with a Markov chain algorithm with 1,000,000 steps and 100,000 dememorization steps.

Following these steps, 17 SNP loci were excluded (Additional file 1: Table S3) before calculating Wright’s *F*-statistics [49] per field and globally for the entire sample, using Arlequin. Pairwise *F*_ST_ values [50] were computed to quantify genetic differentiation between fields. We also did a locus-by-locus analysis of molecular variance (AMOVA) with fields grouped into townships to hierarchically partition genetic variance into regions, fields and individuals.

Mating structure was investigated by computing *F*_IS_ values per field as well as for the entire population. *F*_IS_ was used to estimate the degree of sibmating, following [51-53]:

1 $$ \alpha =4\frac{\left({F}_{\mathrm{IS}}\right)}{\left(3\times {F}_{\mathrm{IS}}+1\right)} $$

The degree of sibmating α was then used to predict the proportion of diploids that was male (DMP). However, the occurrence of diploid males is the result of matings that are matched in terms of sex alleles, and not of sibmatings *per se*. In order to predict DMP, we thus need to predict the frequency of matched matings *θ* first [54]. In a randomly mating population, *θ* depends on sex allele diversity, with a higher sex allele diversity resulting in a lower *θ*. Under inbreeding, *θ* is increased because brothers and sisters share sex alleles at a higher rate, independent of sex allele diversity in the population. Ultimately, *θ* and DMP depend on: (1) the number of complementary sex loci; (2) sex allele diversity at each locus; (3) degree of sibmating α; and (4) diploid male developmental survival relative to that of females. For *C. vestalis*, α is derived from our field study using formula (1), and developmental survival of diploid males appeared to be high in laboratory studies [26], so we will consider it equal to that of females. Laboratory crosses suggest the presence of CSD with two independent loci in *C. vestalis*, but we will also consider sl-CSD for comparison.

Let us first consider sl-CSD. In a randomly mating population with *k* sex alleles at a single sex locus, the frequency of matched matings *θ* is 2/*k* [11,54]. This proportion is much higher for an inbreeding population because half of all matings between sibs are matched and result in the production of diploid males, independent of sex allele diversity. Matched matings result in 50% of diploid offspring developing as males, thus DMP is calculated as follows:

2 $$ DM{P}_{sl-CSD} = 0.25\alpha +\frac{\left(1-\alpha \right)}{k} $$

where α = degree of sibmating and *k* = number of sex alleles.

Under CSD with 2 independent loci (two-locus CSD), on the other hand, diploid males develop from fertilized eggs that are homozygous at both sex loci, while heterozygosity at one or both loci leads to female development. Predicting the proportion of diploid males is thus more complex. In a randomly mating population, *θ* depends on the number of sex alleles at the first locus (*k*) and the second locus (*l*) (for simplicity, we will assume that *k = l*). Matched matings with females that are heterozygous at both loci result in 25% diploid males, while matched matings with females that are homozygous at one locus result in 50% diploid males (effectively the same as sl-CSD). Under inbreeding, half of sibmatings with homozygous females are matched while 1/4^th^ of sibmatings with heterozygous females are matched at both sex loci. Overall, DMP is calculated as follows:

3 $$ DM{P}_{2l-CSD} = 0.0625\cdot \alpha \cdot {f}_{\mathrm{HET}}+0.25\cdot \alpha \cdot \left(1-{f}_{\mathrm{HET}}\right)+0.25\cdot \left(1-\alpha \right)\cdot {p}_{\mathrm{HET}} + 0.5\cdot \left(1-\alpha \right)\cdot {p}_{\mathrm{HOM}} $$

where α = degree of sibmating, *f*_HET_ is the frequency of females heterozygous at both sex loci under random mating, and *p*_HET_ and *p*_HOM_ are the frequency of matched matings involving females heterozygous at both sex loci or homozygous at one sex locus respectively.

## Results

C. vestalis *parasitism and diploid males in Taiwan*

Diamondback moth infestation rates were high in non-commercial cabbage fields in Western Taiwan, with approximately 40 to more than 600 caterpillars per plant (Additional file 1: Table S1). A total of 6,413 *C. vestalis* cocoons were collected and reared from collected hosts, with averages across field collection sites of between 6 and more than 100 per plant (Table 1). Overall parasitism rate of diamondback moth by *C. vestalis* was 0.23 but parasitism was highly variable between fields (between 0.04 and 0.51, Table 1). Average sex ratio of emerged *C. vestalis* was 0.49 ± 0.02 males and sex ratio was similar across fields (0.41 to 0.57, Table 1).

**Table 1:** **Overview of data collected from eight fields in Western Taiwan**

**Field**	**Infestation level**	***C. vestalis***	**Parasitism rate**	**Sex ratio of** ***Cv***	**DMP**
1	73.7 ± 7.7	6.3 ± 1.0	0.09 ± 0.02	0.57 ± 0.08	0
2	639.3 ± 92.9	97.4 ± 12.1	0.15 ± 0.01	0.47 ± 0.02	0.029
3	140.7 ± 14.8	24.1 ± 3.2	0.17 ± 0.02	0.48 ± 0.03	0.005
4	67.0 ± 14.3	9.1 ± 1.5	0.14 ± 0.03	0.41 ± 0.13	0
5	115.0 ± 15.7	49.1 ± 6.4	0.43 ± 0.04	0.44 ± 0.04	0.008
6	40.8 ± 6.7	8.7 ± 1.0	0.21 ± 0.05	0.50 ± 0.06	0
7	165.0 ± 19.2	7.3 ± 1.3	0.04 ± 0.01	0.51 ± 0.09	0.020
8	257.4 ± 30.0	130.3 ± 16.4	0.51 ± 0.03	0.53 ± 0.02	0.010
Total			**0.23 ± 0.08**	**0.49 ± 0.02**	**0.014**

Infestation levels (diamondback moth larvae and *C. vestalis* combined) and numbers of *C. vestalis* (averages per plant ± se), and parasitism rates and sex ratios of *C. vestalis* (averages ± se weighted by infestation level and number of adult *C. vestalis* respectively), and estimated proportion of diploid males (DMP = diploid males/(diploid males + females)).

Analysis of male ploidy levels revealed the presence of 21 diploid males among 1,405 analysed males (Additional file 1: Table S2). Triploids were not found. The proportion of diploid males among all diploids (calculated per field as: diploid males/(diploid males + females), DMP) was thus low in all fields, varying between 0 and 0.03 (Table 1), with an overall DMP of 0.014. Diploidy was confirmed by genotyping at 98 SNPs for all 21 males by the presence of one or more heterozygous SNP loci.

### SNP detection and validation

By aligning individual reads against the *de novo* assembled reference genome of *C. vestalis*, we detected 270,476 putative SNPs across the genome (on average one SNP per 590 bp)(sequence of scaffolds and contigs of reference genome, and information about putative SNPs are available in Dryad [80]. Average depth of sequencing at putative SNP sites was 43X. As a first indication of the validity of our SNP calling procedure, we calculated the ratio of transitions:transversions, which was 2.18:1 for our dataset, well in the range of published ratios [40,41,55,56] and clearly different from the ratio that would be produced by chance (1:2) [57-59].

Of the 100 putative SNPs selected for assay development [80], 98 were polymorphic in the validation panel of eight females. This translates into a SNP conversion rate of 98%, comparing favourably with published studies, because conversion can be as low as around 50% [60] and references therein.

### Genetic structure

The set of 98 polymorphic SNPs was used to study genetic structure of 139 *C. vestalis* females collected in Western Taiwan (Additional file 1: Table S3 and dataset in Dryad [80]. Bayesian clustering without prior information on sampling location suggested that the most likely number of populations in our sample was three (*K* = 3 using Structure and Evanno’s delta *K* method [45]).

We then investigated linkage disequilibrium and deviations from Hardy-Weinberg equilibrium of our SNP marker set to evaluate their suitability as population genetic markers. Individuals of the largest cluster identified by Structure were used for this purpose, using a conservative threshold of 0.9 probability to belong to a specific genetic cluster (76 individuals, Additional file 1: Table S4). Six out of 98 loci deviated significantly from HWE (Additional file 1: Table S3), one of these loci showed heterozygote excess and five were heterozygote deficient. Analysis of linkage disequilibrium indicated significant linkage between 24 (out of 4,753) pairs of loci (after sequential Bonferroni correction) involving 25 loci (Additional file 1: Table S3). Subsequent analyses presented in this paper were done with 81 loci after excluding the 6 loci that deviated from HWE and randomly excluding 11 additional loci so that no pairs of loci were in significant LD anymore (Additional file 1: Table S3). Analyses using the complete set of 98 loci are reported in Additional file 1: (Tables S5, S6 and S7) and revealed no qualitative differences.

**Figure 1:**
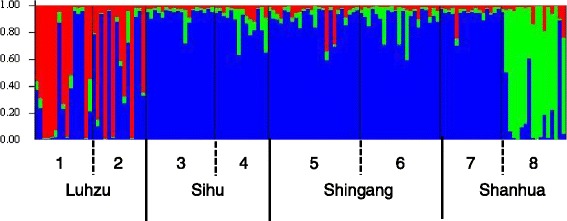
Structure clustering of 139 female *C. vestalis* from eight fields into three genetic clusters. Each individual is represented by a vertical bar, with colours within bars indicating the probability of an individual belonging to cluster A (red), cluster B (blue) or cluster C (green). Numbers on the x-axis indicate fields.

Overall *F*_ST_ was significant at 0.048 (95% CI 0.038-0.059), and pairwise comparisons between fields corresponded with the genetic clusters inferred by Structure (Figure 1 and Table 2). Fields 1 and 2 that comprised individuals assigned to genetic cluster A were significantly different from all the other fields but were not significantly different from each other. Field 8 that comprised individuals assigned to genetic cluster C was also significantly different from all other fields. Pairwise *F*_ST_ values between fields 3–7 were non-significant, except for fields 6 and 7, supporting clustering of the majority of individuals from these fields in cluster B (Additional file 1: Table S4). Hierarchical partitioning of genetic variance using AMOVA indicated a low level of genetic differentiation between townships and between fields within townships. Most of the genetic variation was present among individuals within fields (Table 3). This is not surprising given the largest cluster identified by Structure included the majority of wasps collected from three of four townships. Township thus does not appear to be a good predictor of population structure.

**Table 2:** **Pairwise** ***F***_**ST**_ **for** ***C. vestalis*** **parasitoids collected from eight fields in Western Taiwan**

	**Luhzu1**	**Luhzu2**	**Sihu3**	**Sihu4**	**Shingang5**	**Shingang6**	**Shanhua7**	**Shanhua8**
**Luhzu1**	-	0.106	<0.0001	<0.0001	<0.0001	<0.0001	<0.0001	<0.0001
**Luhzu2**	0.0125	-	<0.0001	0.0001	<0.0001	<0.0001	0.0001	<0.0001
**Sihu3**	**0.0653**	**0.0489**	-	0.091	0.063	0.449	0.036	<0.0001
**Sihu4**	**0.0670**	**0.0517**	0.0078	-	0.162	0.043	0.026	<0.0001
**Shingang5**	**0.0631**	**0.0460**	0.0068	0.0053	-	0.184	0.0021	<0.0001
**Shingang6**	**0.0674**	**0.0498**	0.0005	0.0111	0.0041	-	0.0011	<0.0001
**Shanhua7**	**0.0663**	**0.0443**	0.0112	0.0159	0.0195	**0.0218**	-	<0.0001
**Shanhua8**	**0.1283**	**0.1240**	**0.0852**	**0.0577**	**0.0726**	**0.0642**	**0.0986**	-

*F*_ST_ values (below diagonal) in bold are significant after Bonferroni correction for multiple comparisons (adjusted to 0.0018; values above diagonal are p-values). Analyses are based on 139 female wasps genotyped at 81 SNP loci.

**Table 3:** **Hierarchical partitioning of genetic variance**

	**d.f.**	**Sum of squares**	**Variance components**	**% variance**
Between townships	3	155.113	0.29	1.79
Between fields within townships	4	128.785	0.50	3.06
Within fields	270	4065.522	15.49	95.15
Total	277	4349.419	16.28	

AMOVA of *C. vestalis* collected from 8 fields in four townships in Western Taiwan using 139 female wasps genotyped at 81 SNP loci.

### Mating structure and sex determination load

Over all loci, average heterozygosity varied between 0.348 ± 0.018 in field 1 to 0.420 ± 0.020 in field 7 (Table 4). The highest *F*_IS_, was found in field 1 (*F*_IS_ = 0.111), while fields 3 and 7 had an *F*_IS_ lower than 0 (Table 4). Across all fields, *F*_IS_ was 0.021 (95% CI 0.001 to 0.042). Observed *F*_IS_ and the lower and upper limit of the 95% CI were used to estimate the degree of sibmating α (formula 1), which was 8.1% for the entire population (range 0.0-15.1%).

**Table 4:** **Genetic variation within individuals of** ***C. vestalis*** **in Taiwan**

**Field**	**N**	**Monomorphic loci**	**Obs. Het. ± se**	**Exp. Het. ± se**	***F***_**IS**_
Luhzu1	15	4	0.348 ± 0.018	0.390 ± 0.014	0.111
Luhzu2	14	2	0.394 ± 0.019	0.395 ± 0.013	0.003
Sihu3	18	1	0.402 ± 0.018	0.401 ± 0.014	-0.003
Sihu4	14	4	0.412 ± 0.020	0.417 ± 0.014	0.011
Shingang5	24	0	0.382 ± 0.015	0.393 ± 0.013	0.031
Shingang6	21	1	0.373 ± 0.017	0.387 ± 0.014	0.036
Shanhua7	16	2	0.420 ± 0.020	0.402 ± 0.013	-0.047
Shanhua8	17	6	0.359 ± 0.021	0.370 ± 0.016	0.029
Total	**139**	**0**			**0.021**

Number of wasps, number of monomorphic loci, observed and expected heterozygosity (Mean ± se), and inbreeding coefficient *F*_IS_ of *C. vestalis* collected from eight fields. Analyses are based on 139 female wasps genotyped at 81 SNP loci.

**Figure 2:**
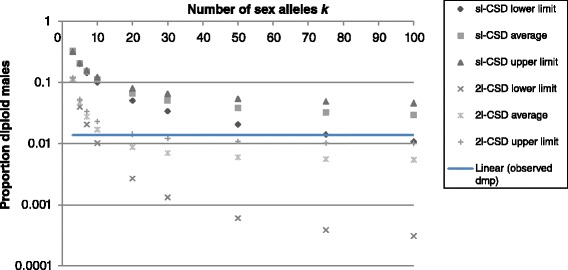
Predicted proportion of diploid males for *C. vestalis* for CSD with one locus (sl-CSD) or two independent loci (2 l-CSD) for sex allele diversity *k* ranging from 3 to 100. We assumed 8.1% sibmating as found in Western Taiwan and diploid male developmental survival to be equal to that of females. We used the 95% confidence interval on the proportion sibmating to calculate upper and lower limits for the predicted proportion diploid males. See text for detailed explanation and formulae used.

Using these values of α, we then predicted DMP in the Taiwanese population of *C. vestalis*, for an sl-CSD and a two-locus CSD scenario (see formulae 2 and 3 in methods), with sex allele diversity between 3 and 100 per locus. The observed DMP was 1.4% in this population of *C. vestalis*. Under CSD with two independent loci, the observed value is within the predicted range when sex allele diversity is between 9–20 alleles at each locus (Figure 2). Under sl-CSD, however, at least 75 sex alleles are required to result in 1.4% diploid males.

## Discussion

Most studies on complementary sex determination have been performed in the laboratory. Yet, studying the genetic load associated with CSD (i.e. diploid males) in relation to mating structure of natural populations allows placing sex determination in an evolutionary context. Mating structure and sex determination are tightly intertwined in hymenopteran insects because the production of diploid males increases dramatically under conditions of inbreeding. Hence, mating systems characterized by inbreeding are thought to select for systems of sex determination with fewer or no diploid males [7]. To date, the best example is found in *Nasonia* parasitoids, which naturally have high levels of sibmating and have no CSD [61]. Instead, sex is determined through maternal imprinting in *N. vitripennis*, which does not lead to the production of diploid males under inbreeding [24,25]. Conversely, in species with CSD, selection is expected to act on factors that minimize the genetic load associated with CSD, i.e. to minimize the production or cost of diploid males. Here, we made a population-level analysis of CSD in a natural population of the solitary parasitoid wasp *Cotesia vestalis* by assessing DMP, and evaluating mating and population structure.

### Diploid males in natural populations

We detected 21 diploid males in our field study in Western Taiwan, and the overall rate of males among diploids was 0.014, which is low compared to other *Cotesia* species. A Swiss population of *C. glomerata* contained diploid males at a rate of 0.06 [62], while DMP was 0.08-0.13 in a North American population of *C. rubecula* [35]. Low DMP was expected in *C. rubecula* based on its two-locus CSD phenotype. However, this species was introduced in North America from its native range in Europe for biological control purposes and a genetic bottleneck might have reduced the sex determination system to effectively sl-CSD, hence resulting in higher rates of diploid males. Similar effects of genetic bottlenecks on DMP have been shown in *Soenopsis invicta* ants and *Polistes chinensis antennalis* wasps [63,64]. Conversely, *C. glomerata* was expected to have higher DMP because it has sl-CSD and a mating system characterized by a relatively high level of inbreeding [32,33]. Low DMP in *C. glomerata* may be explained by post-copulatory processes, such as biased fertilization of eggs with compatible sperm (in terms of sex alleles) [62].

Diploid males arise from matings that are matched in terms of sex alleles, and thus DMP depends on the frequency of matched matings, which in turn depends on the degree of sibmating and sex allele diversity [11,54]. We used the estimated rate of sibmating found in the field to predict DMP for *C. vestalis* for two CSD scenarios - single locus and two independent loci - and a wide range of sex allele numbers (Figure 2). These predictions illustrate that sl-CSD only results in very low DMP when sex allele diversity is very high; even with 100 different sex alleles, 8.1% sibmating still results in DMP of 0.03. Although most empirical estimates of sex allele diversity are in the range of 9–20 different alleles [11], recent *csd* sequence analyses in honeybees dramatically increased estimates of sex allele diversity from 11 to 20 alleles to almost 100 alleles locally ([54,65,66]. Two native populations of the fire ant *S. invicta* also harbour 66–86 sex alleles [63]. These findings suggest that high numbers of sex alleles can be maintained in finite populations despite the effects of random genetic drift [67,68]. Mate choice could also result in lower than expected DMP, for example based on kinship or on sex allele identity [69]. However, lab studies suggest that *Cotesia* females are not particularly choosy when it comes to males and they readily mate with brothers or with diploid males [34,35,70]. Moreover, controlled laboratory crosses, without an effect of mate choice, also resulted in low DMP in *C. vestalis* [26]. When we combine these laboratory studies with the field data collected in the present study, we consider CSD with two independent loci and moderate sex allele diversity a more plausible explanation of low DMP in *C. vestalis* than sl-CSD with high sex allele diversity (Figure 2). Ultimately, genetic mapping of the sex loci and/or sequencing of the sex determination genes in *C. vestalis* is necessary to provide hard evidence for the existence of two-locus CSD.

### Genetic structure

Distinct genotypes were found among wasps collected in Luhzu township (fields 1 and 2), and in field 8 in Shanhua township, as indicated by clustering in Structure and supported by significant *F*_ST_ values between these fields and all other fields (Figure 1 and Table 2). However, the largest cluster of wasps included individuals from all eight fields (Additional file 1: Table S4), and hierarchical partitioning of genetic variance indeed suggests that township and field are no good predictors of population structure in *C. vestalis*. Overall, we found a low level of genetic differentiation between *C. vestalis* from different fields and a high level of variation within fields (AMOVA, Table 3). Similar to our findings in *C. vestalis*, low levels of genetic differentiation between populations and a high level of genetic variation within populations were found for other parasitoids, including *Campoletis sonorensis* and *Chelonus insularis* [71], *Cotesia glomerata* [32], and *Aphidius ervi* [72]. These studies suggest substantial gene flow over long distances, which counters genetic drift within populations. With respect to CSD, while few sex alleles may get lost locally due to genetic drift in such populations, introgression can maintain sex allele diversity at a larger scale. Interestingly, these studies were performed in an agricultural landscape with high frequencies of hosts. In comparison, different results were obtained in other studies on more natural systems of hosts and parasitoids. Global population differentiation of the parasitoid *Neotypus melanocephalus*, which parasitizes caterpillars of the endangered butterfly *Maculinea nausithous* that occur at low densities, was high [73], suggesting limited migration of parasitoids and an effect of small population sizes. Differences in population structure between parasitoid species are probably best explained by a combination of differences in migration rates (dispersal ability) [74] and population sizes, which in turn depend on host densities [73].

### SNP marker development

We showed that SNP mining by *de novo* Illumina sequencing of the entire genome is an efficient way to develop a set of molecular markers for a non-model species. Due to its small genome size, a single lane of paired-end Illumina sequencing was sufficient to enable a near-complete (but fragmented) *de novo* assembly of the *C. vestalis* genome. Because we used pooled DNA of eight *C. vestalis* females, mapping reads against our reference genome allowed for detection of a large number of putative SNPs. Our method is an economical, fast, and qualitatively robust way of developing a genome-wide set of molecular markers for non-model species; for example compared to the rather laborious methods of microsatellite development [75]. A drawback may be the specificity of SNPs for the population they were developed on [76,77], but this may be compensated by the large number of putative SNPs available for assay development. Preliminary tests on a small panel of *C. vestalis* wasps from different populations look promising: the large majority of the set of 98 markers amplified, and 10-45% of the markers was polymorphic in individuals collected from the field in the Netherlands or from laboratory crosses (J. G. de Boer; unpublished data).

## Conclusions

In conclusion, we found a low level of sibling mating in a native population of *C. vestalis* in Taiwan, confirming our expectation of non-random mating. Even though *C. vestalis* is a solitary parasitoid, sibling matings may occur when siblings emerge from different host individuals that are spatially close to each other, for example on the same plant. This seems a likely explanation in *C. vestalis*, because its host, diamondback moth, may reach high densities, especially in agricultural landscapes. Comparisons of mating structure of closely related species parasitizing hosts with different spatial distribution patterns could be informative in this respect. *Cotesia vestalis* indeed produced diploid males at a low rate under natural conditions as we predicted from our laboratory finding of two-locus CSD. Diploid males in *C. vestalis* are developmentally viable and produce infertile triploid offspring [34]. This is considered the most costly scenario of CSD in terms of effects on population growth rate and extinction risk in small populations in comparison to species in which diploid males die during development or evolved fertile diploid males [9,68,78]. We think that the relatively high fitness cost of diploid males, combined with non-random mating may have selected for evolution from sl-CSD to CSD with two independent loci in *C. vestalis*.

## Availability of supporting data

The datasets supporting the results of this article are available in the Short Read Archive of NCBI (raw Illumina reads Accession SRP058413), and Dryad (the assembled scaffolds and contigs, SNP information and sequences, list of putative SNPs and SNP genotypes) http://doi:10.5061/dryad.jn14s, and is partly available within this article as the following additional tables and figure:

Table S1: Overview of *Pl. xylostella* and *C. vestalis* collected per field in Western Taiwan.

Table S2: Flow cytometric analysis of *C. vestalis* male ploidy levels per field.

Table S3: Observed and expected heterozygosity per locus for parasitoid wasps, and linkage between loci.

Table S4: Assignment of individual wasps to three genetic clusters inferred by Structure.

Table S5: Hierarchical partitioning of genetic variance (AMOVA) of *C. vestalis* genotyped at 98 SNP loci.

Table S6: Pairwise *F*_ST_ for *C. vestalis* parasitoids based on genotypes at 98 SNP loci.

Table S7: Observed and expected heterozygosity and *F*_IS_ of *C. vestalis* based on genotypes at 98 SNP loci.

Figure S1: Evanno’s DeltaK plot based on Structure suggesting 3 genetic clusters.

## Additional file

Additional file 1: Table S1. Overview of *Pl. xylostella* and *C. vestalis* collected per field in Western Taiwan. **Table S2.** Flow cytometric analysis of *C. vestalis* male ploidy levels per field. **Table S3.** Observed and expected heterozygosity per locus for parasitoid wasps, and linkage between loci. **Table S4.** Assignment of individual wasps to three genetic clusters inferred by Structure. **Table S5.** Hierarchical partitioning of genetic variance (AMOVA) of *C. vestalis* genotyped at 98 SNP loci. **Table S6.** Pairwise *F*_ST_ for *C. vestalis* parasitoids based on genotypes at 98 SNP loci. **Table S7.** Observed and expected heterozygosity and *F*_IS_ of *C. vestalis* based on genotypes at 98 SNP loci. **Figure S1.** Evanno’s DeltaK plot based on Structure suggesting 3 genetic clusters.
