# Supplementary material for: Population-level consequences of complementary sex determination in a solitary parasitoid
Source: BMC Evol Biol. 2015 May 30;15:98. doi: 10.1186/s12862-015-0340-2 (PMC4461988; doi:10.1186/s12862-015-0340-2)

Additional file with supplementary tables and figure for 'Population-level consequences of complementary sex determination in a solitary parasitoid' by de Boer et al.

Supplementary Table S1: Total number of diamondback moth (DBM) larvae, number of *C. vestalis* cocoons, emerged female and male *C. vestalis* adults, number of cocoons that did not emerge, and hyperparasitoids (i.e. species that parasitize on *C. vestalis*) collected from 8 fields in Western Taiwan.

Location	Field (number of sampled plants)	Total collected	DBM	*C. vestalis* cocoons	Female *Cv*	Male *Cv*	Not emerged	Hyper parasitoid
Luhzu	1(20)	1474	1348	126	40	52	34	0
	2 (16)	10228	8670	1558	613	543	401	1
Sihu	3(20)	2813	2332	481	200	183	98	0
	4 (15)	1005	869	136	65	46	25	0
Shingang	5 (24)	2761	1583	1178	438	340	393	7
	6 (21)	856	673	183	72	73	38	0
Shanhua	7 (20)	3299	3154	145	49	52	44	0
	8 (20)	5148	2542	2606	686	772	658	488
TOTAL	**(156)**	**27584**	**21171**	**6413**	**2163**	**2061**	**1691**	**496**

Supplementary Table S2: Flow cytometric analysis of male ploidy level, reported per field, with numbers of *C. vestalis* males in each category (haploid, diploid, unknown ploidy and not analysed), and estimated proportion of diploid males among all diploids (DMP).

Field	Haploid	Diploid	Unknown	Not analysed	DMP
1	45	0	2	3	0
2	377	13	2	193	0.029
3	179	1	2	1	0.005
4	44	0	1	1	0
5	283	3	8	48	0.008
6	68	0	1	2	0
7	49	1	2	0	0.020
8	318	3	3	447	0.010
TOTAL	**1363**	**21**	**21**	**695**	**0.014**

Supplementary Table S3: Observed and expected heterozygosity per locus for parasitoid wasps in cluster B (inferred by STRUCTURE: 76 individuals with probability >0.90) and P-values. Loci in bold have P<0.05 and were removed for further analysis. Additionally, 11 loci in grey font were removed based on analysis of linkage disequilibrium: the last column shows the locus or loci that were linked significantly with the locus in the first column. After excluding loci based on HWE and LD, the remaining set of 81 loci (normal font) was used for further analyses.

Locus	#Genot	H_{obs}	H_{exp}	P-value	Linked loci
**Cv4_10207571**	**75**	**0.253**	**0.346**	**0.037**	
Cv4_103242832	76	0.355	0.449	0.077	
Cv4_104618491	75	0.467	0.494	0.645	
Cv4_114769212	74	0.378	0.325	0.274	
Cv4_11518236	76	0.224	0.277	0.104	
Cv4_115463175	76	0.197	0.200	1.000	
Cv4_115788	76	0.526	0.495	0.643	
Cv4_117070287	76	0.289	0.268	0.680	
Cv4_11747690	74	0.608	0.492	0.057	
Cv4_12825955	76	0.263	0.303	0.260	
Cv4_129597641	76	0.342	0.364	0.750	
Cv4_12966778	74	0.446	0.496	0.480	Cv4_29422884
					Cv4_3533602
					Cv4_82721417
					Cv4_95155918
**Cv4_13002270**	**75**	**0.147**	**0.202**	**0.044**	
Cv4_14949914	73	0.397	0.503	0.100	Cv4_2089441
					Cv4_82721417
Cv4_16464971	71	0.338	0.319	1.000	Cv4_2089441
					Cv4_46090709
Cv4_17187968	75	0.147	0.159	0.433	
**Cv4_1812017**	**76**	**0.303**	**0.465**	**0.003**	
Cv4_18182918	75	0.493	0.474	0.808	
Cv4_184509	76	0.408	0.371	0.535	
Cv4_18943653	74	0.392	0.333	0.168	
Cv4_19013627	71	0.380	0.478	0.132	
Cv4_2001314	76	0.447	0.444	1.000	Cv4_72915572
Cv4_20318802	76	0.474	0.503	0.651	
Cv4_2089441	73	0.452	0.479	0.631	
Cv4_21503731	75	0.307	0.330	0.501	
Cv4_21683215	76	0.487	0.472	0.811	
Cv4_22028866	76	0.408	0.501	0.112	
Cv4_22393	75	0.213	0.213	1.000	
Cv4_23208637	76	0.118	0.157	0.082	
**Cv4_2358512**	**76**	**0.184**	**0.268**	**0.015**	
Cv4_23820790	74	0.527	0.492	0.636	Cv4_3533602
					Cv4_84921037
Cv4_23962912	76	0.184	0.230	0.108	
Cv4_24496635	73	0.247	0.238	1.000	

Cv4_25653070	74	0.446	0.403	0.400	
Cv4_26644668	76	0.447	0.461	0.806	
Cv4_26767185	75	0.547	0.486	0.341	Cv4_3533602<br>Cv4_83078557
Cv4_27616871	73	0.178	0.207	0.236	Cv4_767470
Cv4_2789545	74	0.514	0.485	0.638	
Cv4_29113458	73	0.425	0.490	0.335	
Cv4_29422884	72	0.472	0.465	1.000	
Cv4_33493654	75	0.307	0.280	0.677	
Cv4_34423402	76	0.487	0.501	0.821	
Cv4_3533602	75	0.227	0.297	0.051	Cv4_71660500
Cv4_36580149	76	0.500	0.468	0.624	
Cv4_3802289	75	0.400	0.471	0.221	
Cv4_38841293	75	0.547	0.480	0.331	Cv4_41631235<br>Cv4_71660500<br>Cv4_84921037
Cv4_41631235	75	0.533	0.496	0.640	
Cv4_42188166	75	0.173	0.202	0.226	
Cv4_44822582	75	0.520	0.490	0.640	
Cv4_46014805	76	0.500	0.498	1.000	
Cv4_46090709	72	0.569	0.468	0.080	
Cv4_48622495	76	0.434	0.493	0.350	
Cv4_53711193	73	0.342	0.337	1.000	
Cv4_55495260	76	0.118	0.112	1.000	
Cv4_55697371	75	0.027	0.026	1.000	
Cv4_58796376	74	0.432	0.467	0.615	
Cv4_59924679	76	0.263	0.249	1.000	
Cv4_63452468	74	0.405	0.490	0.156	
Cv4_64296712	76	0.329	0.384	0.233	
Cv4_65607962	70	0.486	0.497	1.000	
Cv4_6630586	76	0.408	0.371	0.533	
Cv4_67446535	76	0.237	0.249	0.646	
Cv4_67732797	76	0.158	0.168	0.482	
Cv4_70224753	75	0.427	0.394	0.560	Cv4_82721417
Cv4_70592452	73	0.370	0.490	0.052	
Cv4_70978951	76	0.487	0.499	1.000	
Cv4_71660500	75	0.587	0.499	0.164	
Cv4_72915572	75	0.520	0.500	0.818	
Cv4_74460715	76	0.158	0.168	0.482	
Cv4_754508	73	0.493	0.503	1.000	
Cv4_75909440	75	0.493	0.468	0.804	
Cv4_767470	74	0.338	0.349	0.746	
Cv4_76928130	76	0.500	0.498	1.000	
Cv4_77410309	69	0.493	0.448	0.430	
Cv4_77612797	75	0.440	0.452	1.000	Cv4_71660500<br>Cv4_84921037

Cv4_77905622	74	0.527	0.446	0.187
Cv4_78766817	76	0.474	0.481	1.000
Cv4_81188101	75	0.400	0.483	0.152
Cv4_81288100	75	0.493	0.452	0.453
Cv4_81385826	76	0.355	0.408	0.268
Cv4_82604228	75	0.333	0.387	0.239
Cv4_82721417	73	0.370	0.352	1.000
Cv4_83078557	74	0.446	0.436	1.000
Cv4_84633979	70	0.371	0.434	0.270
Cv4_84921037	75	0.507	0.488	0.813
Cv4_8496510	76	0.342	0.350	1.000
Cv4_8681723	74	0.365	0.403	0.560
**Cv4_88158210**	**75**	**0.227**	**0.314**	**0.024**
Cv4_88551678	75	0.320	0.417	0.053
Cv4_8953085	76	0.513	0.484	0.639
Cv4_90144291	70	0.400	0.398	1.000
Cv4_92102194	73	0.479	0.494	0.816
Cv4_95155918	75	0.533	0.488	0.481
Cv4_9602440	75	0.213	0.192	1.000
Cv4_962560	76	0.408	0.484	0.231
**Cv4_97022122**	**76**	**0.618**	**0.478**	**0.015**
Cv4_9862045	75	0.520	0.474	0.468
Cv4_9966888	71	0.183	0.190	0.562

Supplementary Table S4: Assignment of individual wasps to three genetic clusters inferred by STRUCTURE. The threshold of probability of cluster assignment was set at 0.9. Individuals with <0.9 probability of belonging to any of the three clusters are grouped in "Mix".

Field	A	Genetic cluster B	C	Mix
1	6	3	0	6
2	3	4	0	7
3	0	16	0	2
4	0	7	0	7
5	0	17	0	7
6	0	14	0	7
7	0	14	0	2
8	0	1	8	8
TOTAL	**9**	**76**	**8**	**46**

Supplementary Table S5: Hierarchical partitioning of genetic variance (AMOVA) of *C. vestalis* genotyped at 98 SNP loci. Results of 139 female wasps collected from eight fields in four townships in Western Taiwan.

	d.f.	Sum of squares	Variance components	% variance
Between townships	3	180.48	0.34	1.74
Between fields within townships	4	149.10	0.55	2.81
Within fields	270	4926.67	18.77	95.46
TOTAL	277	5256.23	19.66	

Supplementary Table S6. Pairwise F_{ST} for *C. vestalis* parasitoids collected from eight fields in Western Taiwan (below diagonal); F_{ST} values in bold are significant after Bonferroni correction for multiple comparisons (adjusted to 0.0018; values above diagonal are p-values). Analysis is based on 139 female wasps were genotyped at 98 SNP loci.

	Luhzu1	Luhzu2	Sihu3	Sihu4	Shingang5	Shingang6	Shanhua7	Shanhua8
Luhzu1	-	0.118	<0.0001	<0.0001	<0.0001	<0.0001	<0.0001	<0.0001
Luhzu2	0.0115	-	<0.0001	0.0001	<0.0001	<0.0001	0.0001	<0.0001
Sihu3	**0.0551**	**0.0383**	-	0.254	0.092	0.471	0.009	<0.0001
Sihu4	**0.0576**	**0.0429**	0.0026	-	0.194	0.033	0.004	<0.0001
Shingang5	**0.0600**	**0.0395**	0.0059	0.0046	-	0.298	0.0002	<0.0001
Shingang6	**0.0650**	**0.0423**	0.0004	0.0117	0.0027	-	0.0006	<0.0001
Shanhua7	**0.0717**	**0.0446**	0.0151	0.0230	**0.0212**	**0.0234**	-	<0.0001
Shanhua8	**0.1153**	**0.1115**	**0.0756**	**0.0557**	**0.0695**	**0.0604**	**0.0944**	-

Supplementary Table S7. Number of monomorphic loci, observed and expected heterozygosity (Mean ± se), and inbreeding coefficient F_{IS} of *C. vestalis* collected from eight fields in Western Taiwan. Analyses based on 139 female wasps genotyped at 98 SNP loci.

Field	N	Monomorphic loci	Obs. Het. ± se	Exp. Het. ± se	F_{IS}
Luhzu1	15	5	0.353±0.017	0.394±0.013	0.107
Luhzu2	14	2	0.390±0.018	0.389±0.013	-0.003
Sihu3	18	1	0.399±0.016	0.400±0.012	0.004
Sihu4	14	4	0.403±0.019	0.407±0.013	0.009
Shingang5	24	0	0.378±0.014	0.394±0.012	0.041
Shingang6	21	1	0.373±0.016	0.390±0.012	0.047
Shanhua7	16	2	0.411±0.019	0.395±0.013	-0.042
Shanhua8	17	7	0.368±0.019	0.377±0.014	0.026
TOTAL	**139**	**0**			**0.026**

Supplementary Figure S1: Evanno's DeltaK plot based on STRUCTURE with the set of 139 females and 98 markers, showing the delta K peak at K=3, thus suggesting 3 genetic clusters.

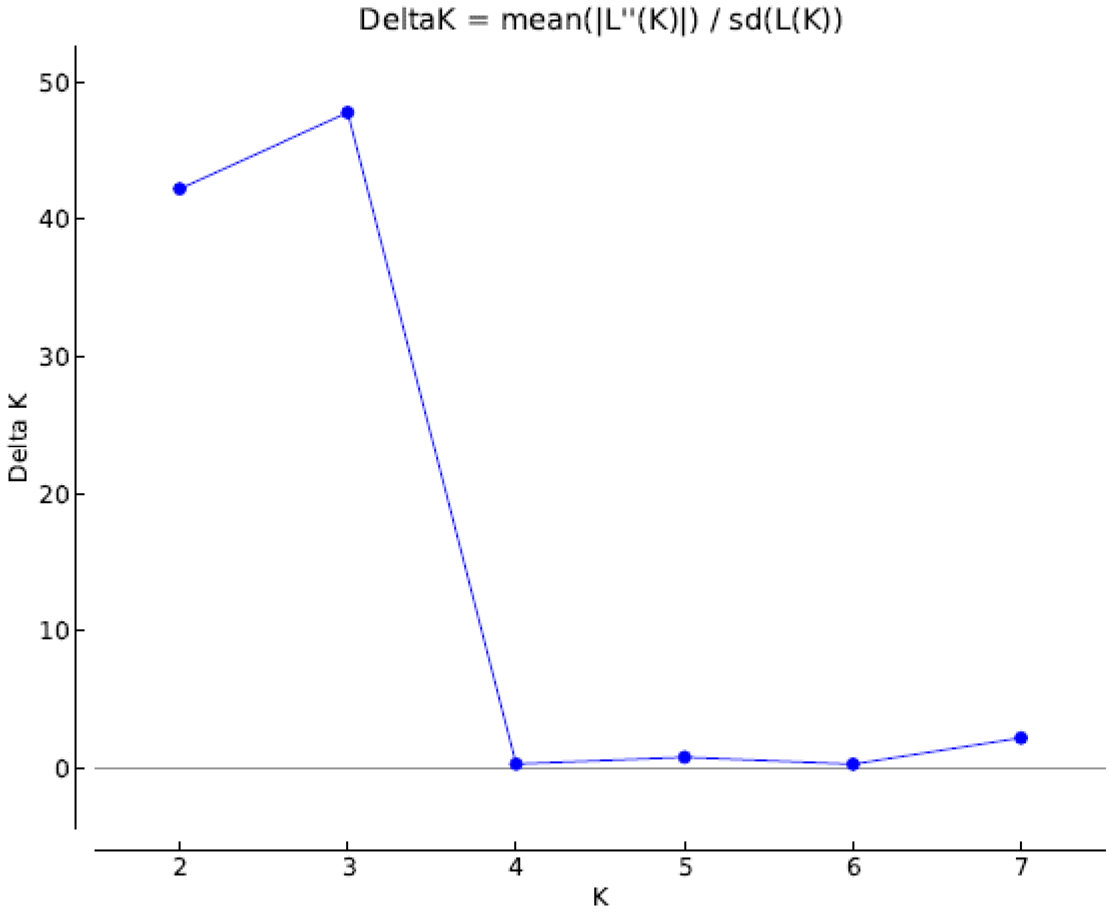

Supplement: Additional file 1: Table S1. — Overview of Pl. xylostella and C. vestalis collected per field in Western Taiwan. Table S2. Flow cytometric analysis of C. vestalis male ploidy levels per field. Table S3. Observed and expected heterozygosity per locus for parasitoid wasps, and linkage between loci. Table S4. Assignment of individual wasps to three genetic clusters inferred by Structure. Table S5. Hierarchical partitioning of genetic variance (AMOVA) of C. vestalis genotyped at 98 SNP loci. Table S6. Pairwise F ST for C. vestalis parasitoids based on genotypes at 98 SNP loci. Table S7. Observed and expected heterozygosity and F IS of C. vestalis based on genotypes at 98 SNP loci. Figure S1. Evanno’s DeltaK plot based on Structure suggesting 3 genetic clusters. [file 12862_2015_340_MOESM1_ESM.pdf]
